# Assessment of fractures in endodontically treated teeth restored with and without root canal posts using high-resolution cone beam computed tomography

**DOI:** 10.4317/jced.56854

**Published:** 2020-06-01

**Authors:** David Aristizabal-Elejalde, Luis-Ernesto Arriola-Guillén, Aron Aliaga-Del Castillo, Gustavo-Armando Ruíz-Mora, Yalil-Augusto Rodríguez-Cárdenas

**Affiliations:** 1 Lecturer, Part-time, Division of Oral and Maxillofacial Radiology, School of Dentistry, Universidad Científica del Sur, Lima, Perú; 2 Associate Professor, Division of Orthodontics and Division of Oral and Maxillofacial Radiology, School of Dentistry, Universidad Científica del Sur, Lima, Perú; 3 Lecturer, Part-time, Department of Orthodontics. Bauru Dental School. University of São Paulo, Brazil; 4 Associate Professor, Division of Orthodontics, Faculty of Dentistry, Universidad Nacional de Colombia, Bogotá D.C, Colombia; 5 Associate Professor, Division of Oral and Maxillofacial Radiology, Faculty of Dentistry, Universidad Nacional de Colombia, Bogotá D.C, Colombia

## Abstract

**Background:**

Root fractures are a diagnostic challenge for dentists in endodontic treatment. This study aimed to determine the relationship between the characteristics of tooth fractures and the presence of root canal posts in endodontically treated teeth using high-resolution cone beam computed tomography (CBCT).

**Material and Methods:**

Fifty high-resolution CBCT scans of endodontically treated teeth with a diagnosis of fracture were obtained, of which 30 were from women and 20 were from men. These scans were acquired with three Veraviewepocs 3D units and one 3D Accuitomo 170 unit, with a 40 × 40-mm field of view and 125 μm voxel size. The variables assessed included the type of fracture, extent of fracture, type of retention, post length, cause of endodontic failure, location of the lesion, and time required to detect the fracture (difficulty score). For data analysis, the chi-squared test, Student’s t-test, and multiple linear regression (α <0.05) were used.

**Results:**

No association was found between the type of fracture and type of retention or between the type of fracture and its extent (*P*>0.05). On the other hand, the type of fracture significantly influenced the time required for its detection. Additionally, the most difficult plane for detecting the fracture and the difficulty score were associated, with statistically significant results.

**Conclusions:**

The type of fracture in endodontically treated teeth was not associated with the type of post used for restoration. All of the CBCT systems used to detect tooth fracture showed the same efficiency.

** Key words:**Cone-Beam CT, Tooth Fracture, Root Canal Post.

## Introduction

Root fractures are a diagnostic challenge for dentists because their signs and symptoms are not present in all cases, and they are difficult to differentiate from endodontic treatment failures. Additionally, they cannot always be detected with conventional radiographs (1,2). The etiology of fractures is multifactorial; these factors can be divided into predisposing and iatrogenic factors (3).

Three factors are important to classify root fractures: origin, location, and pattern (4). In their study with cone beam computed tomography (CBCT), Doğan *et al.* (5) found that the additional presence of crown fractures has a negative effect on the spontaneous healing of root fractures. Most teeth extracted due to root fracture were teeth with prosthetic restorations after root canal treatment and dental fillings (6). Additionally, for future implant placement, the early detection of the fractured root after tooth extraction is important to ensure alveolar bone integrity (1).

CBCT images are more effective than periapical radiographs to detect vertical root fractures in teeth with and without endodontic treatment. The maximum accuracy, sensitivity, and specificity have been reported as 96.6%, 93.3%, and 100%, respectively (7). CBCT can be an ideal alternative in the diagnosis of root fracture in endodontics (8-10).

It has been reported that CBCT images have a higher reliability and validity than do intraoral radiographic techniques in managing an endodontic diagnosis and its complications (11,12). Karabucak *et al.* (13) reported an overall incidence of missed canals of 23.04% and found a significant difference in the prevalence of apical periodontitis when one canal was left untreated (*P* <0.05).

It is thought that missing additional canals when using imaging techniques can lead to unsuccessful treatments (14). The prevalence of apical lesions is high and increases in the presence of defective coronal restorations and procedural errors in endodontics, of which incomplete filling was the most common, followed by partial lateral condensation and canals without radiopaque filling (15). Among the main causes of root canal treatment failure are those that could be visualized radiographically, such as open apices, root perforations, and root fractures (16), while 45.1% of endodontically treated teeth showed a periapical lesion when assessed by CBCT (17).

Using high-resolution CBCT images is an alternative for a more reliable diagnosis of root fractures in endodontically treated teeth due to its high accuracy by using the high-resolution/standard (HI-STD) protocol, which reduces the radiation dose (18). To detect root perforations, the high-resolution mode provides significantly greater accuracy than the low-resolution mode (19). In comparison with the results of ex vivo cross-sections (gold standard), in the general range of correct diagnosis with CBCT, the accuracy increased from 92% to 100% within each assessment parameter, and the diagnoses obtained with 3D Accuitomo 170 were as accurate or more accurate than other systems (20).

Additionally, vertical root fractures were significantly more common in teeth with a post (16.2% prevalence) than without a post (1.2%) according to Maddalone *et al.* (21). The sensitivity and specificity of the area under the receiver operating characteristic (ROC) curve of the scan with posts and without posts were significantly higher than those of periapical radiography under the same conditions (22).

Considering the results described in the literature regarding the high diagnostic efficiency of high-resolution tomographic images to detect root fractures in teeth with posts, the purpose of the present study was to determine the relationship between the characteristics of tooth fractures and the presence of root canal posts in endodontically treated teeth using high-resolution cone beam computed tomography scans acquired in four radiology centers.

## Material and Methods

The present retrospective study was approved by the Ethics and Research Committee of the Southern Scientific University, Lima, Peru (Protocol number 268-2018-POS8). The minimum sample size was determined using the online sample calculation software from www.fisterra.com with a 95% confidence level.

**Figure 1:**
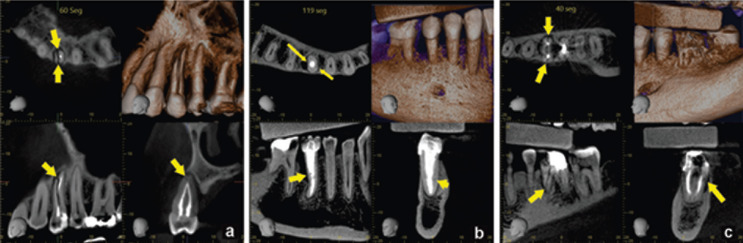
Type of retention: Without post (a), Non-metal post (b), Metal post (c).

**Figure 2:**
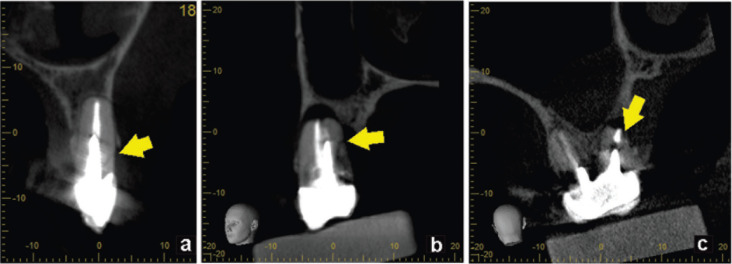
Extent of the fracture: First third (a), middle third (b), apical third (c).

**Figure 3:**
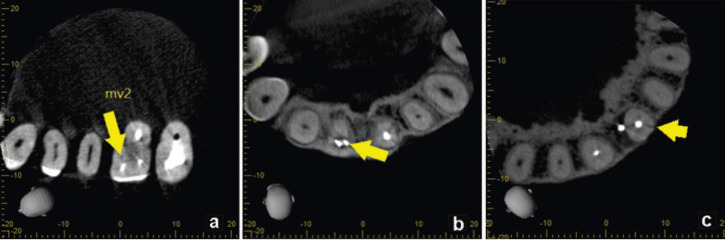
Cause of endodontic failure: Unfilled root canal (a), incompletely filled canal (b), false canal (c).

Fifty high-resolution CBCT scans with a diagnosis of root fracture in endodontically treated teeth were obtained of which 30 (60%) were from women, with a mean age of 55.7 ± 12 years, and 20 (40%) were from men, with a mean age of 47.5 ± 10 years. These scans were acquired in three Veraviewepocs 3D units (J Morita Corp., Kyoto, Japan) and one 3D Accuitomo 170 unit (J Morita Corp., Kyoto, Japan). The size of the field of view (FOV) in all cases was 40 x 40 mm, with a 125 μm voxel size. The exposure factors varied between 80-90 kVp and 6-7 mA, with an approximate dose of 20 μSv per case. All volumes were assessed with i-Dixel 2.0 software (J Morita Corp.). For fracture localization, an iMac 4K iMac display was used with a resolution of 4096 x 2160 pixels (Apple Inc., USA). The time it took the specialist to detect each fracture in the three spatial planes (axial, coronal, and sagittal) in each CBCT was recorded. This measurement was then applied to a categorical scale where different values were matched to the difficulty score: low, up to 60 seconds; medium, from 61 to 180 seconds; and high, above 181 seconds. Subsequently, the radiographic findings were assessed and classified according to the following variables: 1. type of fracture: horizontal, oblique, or vertical (23); 2. Type of retention: without a post, with a non-metal post, with a metal post (Fig. 1); 3. Extent of the fracture: crown, first third of the root canal, middle third of the root canal, or apical third of the root canal (Fig. 2); 4. Cause of endodontic failure: without failure, unfilled root canal, partially filled canal, or false canal (Fig. 3); and 5. Location of the lesions: no lesion, apical lesion, periradicular lesion, pararadicular lesion, or interradicular lesion. All data were recorded in an Excel Table (Microsoft Corp., USA).

In total, 50 CBCT scans were assessed by an oral and maxillofacial radiologist with over 10 years of experience in the interpretation of 3D images acquired with cone beam computed tomography. An intra-examiner assessment was performed for 30% of the samples, with a difference of one month between the first and second assessments to evaluate reproducibility and measure the study error using the Kappa index. This process yielded an agreement value greater than 0.7 for all the categorical variables (Table 1). In the time required to detect the fracture variable, the intraclass correlation coefficient (ICC) was 0.979, with a 95% confidence interval of 0.936 - 0.993.

**Table 1:**
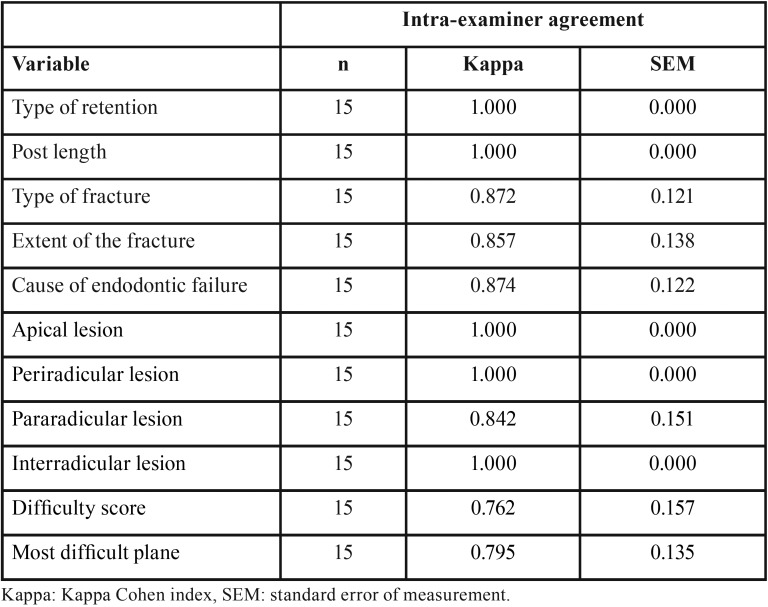
Results of the intra-examiner test for all categorical variables.

-Statistical Analyses

The data analysis was performed using IBM SPSS Statistics 25.0 (Armonk, NY. USA). The results are presented in Tables with descriptive summary measures.

The chi-squared test was used to evaluate the association between the two main categorical variables; a multiple linear regression model was then applied to assess the influence of the variables affecting the time required to detect the fracture. Finally, Student’s t-test was used to compare whether the time required to detect the fracture was different for the two tomography units that were used. A significance level of *P* <0.05 was applied.

## Results

Horizontal fracture was the least common, with 6 cases, followed by vertical fracture with 18 cases. Oblique fracture was the most common, with 26 cases. According to the type of retention, 40% of the sample had no post, 56% had a metal post, and 4% had a non-metal post. Regarding the extent of the fracture, 4% reached the crown, 24% reached the first third of the root canal, 54% reached the middle third, and 18% reached the apical third. In relation to endodontic failures, different causes were found in 38% of the cases, such as unfilled root canals (10), incompletely filled canals (8), and a false canal (1). Regarding the location of the lesion, apical fracture was the most common and was found in 40% of the cases.

There were no significant differences by age and sex; no statistically significant association was found between the type of fracture and type of retention (Table 2) or between the type of fracture and the extent of the fracture (Table 3).

**Table 2:**
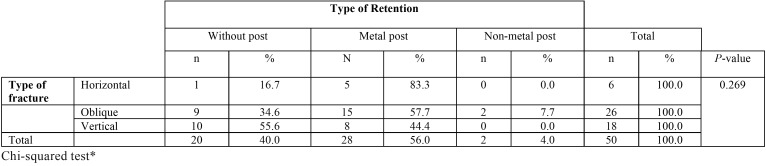
Association between the type of fracture and type of retention.

**Table 3:**
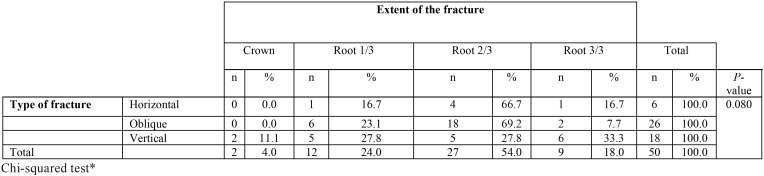
Association between the type of fracture and extent of the fracture.

The endodontic failures that were found did not show a statistically significant association (Table 4). Pararadicular lesions had a statistically significant association with the type of fracture (*P* <0.05) (Table 5).

**Table 4:**
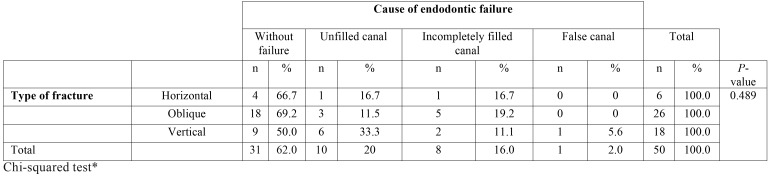
Association between the type of fracture and the cause of endodontic failure.

**Table 5:**
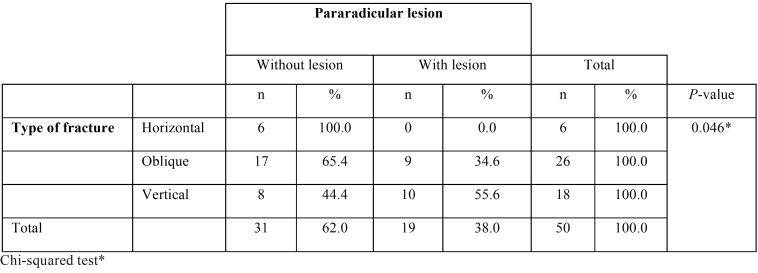
Association between the type of fracture and pararadicular lesion.

The time required to detect the fractures had a statistically significant influence on the detection of oblique and vertical fractures; the time required was 34 seconds less for both types than for horizontal fractures. For fractures in which an interradicular lesion was not present, the time required was 88 seconds longer than for those with this type of lesion (Table 6). There were no statistically significant differences between the times required to detect the fracture according to the CBCT system that was used (Table 7). The most difficult plane for detecting the fracture and the difficulty score were associated, showing statistically significant results (*P* <0.05) ( Table 8).

**Table 6:**
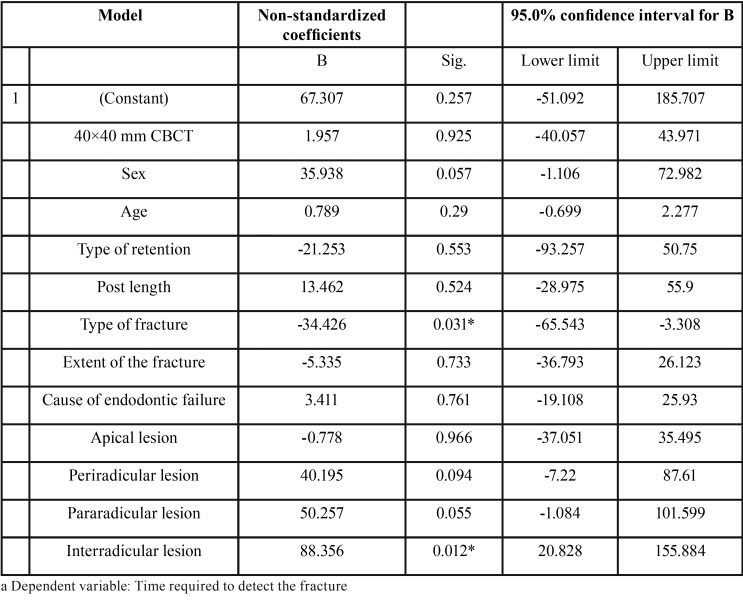
Multiple linear regression to estimate the time required to detect the fracture.

**Table 7:**
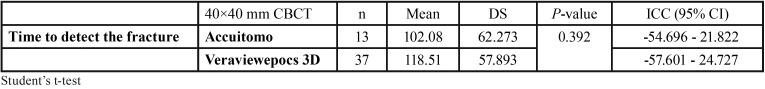
Comparison of the time required to detect the fracture between the two CBCT systems.

**Table 8:**
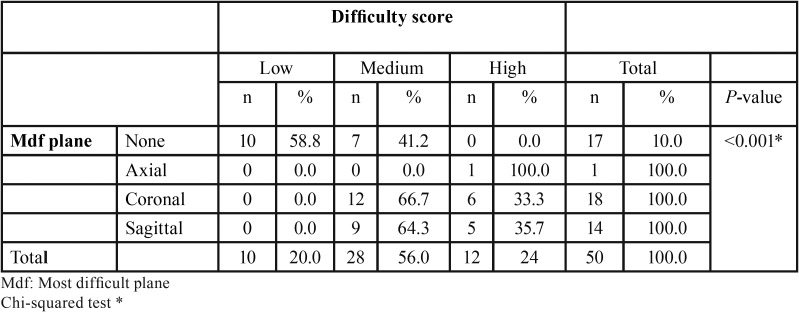
Association between the most difficult plane to detect a fracture and the difficulty score.

## Discussion

In the present study, 50 high-resolution CBCT scans were assessed in patients with a diagnosis of tooth fracture. The results showed that the type of fracture is not related to the type of retention used, which coincides with the results reported by Tsai *et al.* (24) where the presence of a post was not closely related to the presence of horizontal or vertical root fractures in posterior teeth (25). These results contrast with those reported by Maddalone *et al.* (21) in a prevalence study showing an increased risk of vertical root fracture (VRF) in teeth restored with a post; these authors found a statistically significant difference in relation to teeth without a post. In their study, the sample included 61 teeth with a post and VRF and 7 teeth without a post and VRF; more than half of the cases were diagnosed during apical surgery. Additionally, no association was found between the type of fracture and the extent of the fracture, which is in contrast to Huang *et al.* (26) who assessed vertical root fractures greater than 100 microns using micro CT and found that they reached the apical third. The investigation by Huang *et al.* was an *in vitro* study of 37 extracted molars and premolars, while in the present study we included teeth from the 4 quadrants of the oral cavity. This aspect could have had a positive influence on our results.

Regarding the association between the type of fracture and the different causes of endodontic failures, some authors mention the great difficulty of differentiating the signs and symptoms between a lesion caused by root fracture and chronic apical periodontitis caused by endodontic failure (27,28). In our sample, there were many unfilled MB2 canals, incompletely filled canals, and one false canal. However, although we did not find a correlation between them, there were existing lesions that could have originated before the root fracture occurred (29). The present study explored the association between the type of fracture and the location of the lesion, finding that most cases had at least one type of lesion, coinciding with the results of a similar report (30) in which the authors found that the radiolucent J-shaped (halo) image was significantly more common in those cases with vertical root fractures than in controls with no fractures. Arx *et al.* (31) found that in teeth with vertical root fractures, the most common radiographic finding was a periapical lesion, while in the present study, pararadicular lesions were the most common in this type of fracture and were not present in any horizontal fracture, possibly because most vertical fractures extended to the middle third of the root and not to the apical third.

The time required to detect the fracture had a statistically significant influence on detecting oblique and vertical fractures, as this was 34 seconds less than for horizontal fractures. This finding is in contrast to the study by Kamburoglu *et al.* (32), who reported that the radiographic detection of HRF with CBCT is easier than for vertical root fractures. They noted that artifacts caused by root canal filling, pins and posts can complicate the assessment of VRF in fractures in which there is no interradicular lesion, which took 88 seconds longer than in those which did not have this lesion. This measurement was then applied to the difficulty score, finding that most fractures were detected with a medium difficulty score (between 61 and 180 seconds). Notably, all teeth were endodontically treated, and 56% of the sample was restored with a metal post, which was a diagnostic challenge for the radiologist because the artifacts generated by the metal and filling material could affect the time required to detect the fracture. As mentioned by other researchers, the presence of posts and gutta-percha reduces the sensitivity and accuracy in detecting vertical root fractures (32). Other author has reported protocols with smaller FOVs and voxels and achieved better sensitivities and specificities when detecting horizontal root fracture (33).

A comparison between the two tomography systems used showed no significant variation in the time required to detect the fracture. This could be related to the use of equipment from the same manufacturer and using the same voxel size and image analysis software. This coincides with other authors (32) that have used CBCT systems with limited FOVs and high resolution, which behaved similarly in the detection of ex vivo-simulated HRF. According to Metzca *et al.* (34), the highest diagnostic accuracy rate for detecting vertical root fractures was achieved using CBCT with 80 μm voxels (3D Accuitomo 170; CA, USA), as opposed to that reported by Ma *et al.* (35), who did not find significant differences between 80 μm and 125 μm voxel subgroups (*P*=0.320). To improve the accuracy of root fracture detection, other authors have suggested that more evidence is needed regarding the impact of voxel size variation on the diagnostic result in dentistry (36).

The three-dimensional study included the coronal and sagittal planes, which caused the greatest difficulties in detecting the tooth fracture. The plane was one of the elements that changed the difficulty score (*P* <0.05), as BL and MD fractures can more easily be detected in the axial plane because it is perpendicular to the fracture. In other study, the axial plane was found to be the most effective for confirming specific diagnoses, such as root fractures, compared with the coronal and sagittal planes (8). Future studies can use the method proposed, using a difficulty score to measure the time required to detect fractures, making comparisons with smaller voxels and using filters. The method can also be used for other topics of interest.

## Conclusions

The type of fracture in endodontically treated teeth was not associated with the type of post used for restoration. The time required to detect the fracture was less for detecting vertical and oblique fractures than for horizontal ones. The detection time was also lower for fractures in which an interradicular lesion was present. According to the difficulty score, the coronal and sagittal planes presented the most difficulty when trying to detect a root fracture. The efficiency of tooth fracture detection was similar with the two CBCT systems that were used.
